# Non-canonical Estrogen Signaling in Endocrine Resistance

**DOI:** 10.3389/fendo.2019.00708

**Published:** 2019-10-16

**Authors:** Prathibha Ranganathan, Namratha Nadig, Sughosha Nambiar

**Affiliations:** Centre for Human Genetics, Bengaluru, India

**Keywords:** estrogen receptor, tamoxifen, membrane signaling, endocrine resistance, non-genomic actions, selective agonists

## Abstract

Breast cancer is one of the leading causes of cancer related deaths in women worldwide. The disease is extremely heterogenous. A large percentage of the breast cancers are dependent on estrogen signaling and hence respond to endocrine therapies which essentially block the estrogen signaling. However, many of these tumors emerge as endocrine resistant tumors. Many mechanisms have been proposed to explain the emergence of endocrine resistance, which include mutations in the estrogen receptors, cross-talk with other signaling pathways, cancer stem cells etc. This review is focused on the role of non-canonical estrogen receptor signaling in endocrine resistance. Most of the therapeutics which are used currently are targeting the major receptor of estrogen namely ER-α. Last two decades has witnessed the discovery of alternate forms of ER-α, as well as other receptors for estrogen such as ERRgamma, GPER-1 as well as ER-β, which are activated not only by estrogen, but also by the therapeutic agents such as tamoxifen that are routinely used in treatment of breast cancer. However, when the alternate receptors are activated, they result in activation of membrane signaling which subsequently activates pathways such as MAPK and GPCR leading to cell-proliferation. This renders the anticipated anti-estrogenic effects of tamoxifen less effective or ineffective. Future research in this area has to focus on the alternate mechanisms and develop a combinatorial strategy, which can complement the existing therapeutics to get better outcome of endocrine therapies.

## Introduction

Breast cancer is an extremely heterogenous malignancy and a leading cause of cancer related deaths throughout the world. A large percentage of the breast cancers are estrogen sensitive and respond well to endocrine therapy. This mode of therapy essentially blocks the major proliferative pathway namely Estrogen Receptor (ER) signaling. The major strategies for doing this are

1. Using Selective Estrogen Receptor Modulators (SERMS, Ex: Tamoxifen) to block the binding of estrogen to ER
2. Down regulating the receptor using Selective Estrogen Receptor Down-regulators (SERD, Ex: Fulvestrant)
3. Reducing the synthesis of estrogen using aromatase inhibitors.

For a long time, use of SERMs had been very popular. Despite showing very good effects on ER positive tumors, a large percentage of tumors developed resistance to this mode of treatment. Clinicians and researchers have been trying to understand the basis of this resistance to improvise on the treatment strategies. Many mechanisms have been proposed for the development of endocrine resistance. This includes mutations in the ER-α, cross talk with other growth factor pathways etc. (1). This article tries to summarize some of the mechanisms, namely the role of alternative forms of ER-α, ER-β, and other receptors for estrogen such as GPER-1 in development of endocrine resistance.

In humans, the endogenous estrogens are estrone (E1), estradiol (E2), and estriol (E3). Among these, estradiol (E2) is the most prevalent and potent. The main actions of estrogens are mediated by the estrogen receptor (ER) which belongs to the family of nuclear hormone receptors. In the classical model for steroid hormone signaling, the hormone enters the cells through the plasma membrane and binds to the compatible receptor which is mostly localized in the cytoplasm. This binding often leads to dimerization followed by nuclear localization. Once in the nucleus, they bind directly to the DNA response elements such as ERE and regulate transcription of target genes, which in turn alters the biological response of the cells. In an alternate mechanism, the receptors do not bind directly to DNA, but still regulate transcription by forming a complex with co-activators or co-repressors [reviewed in (2, 3)].

**Figure 1:**
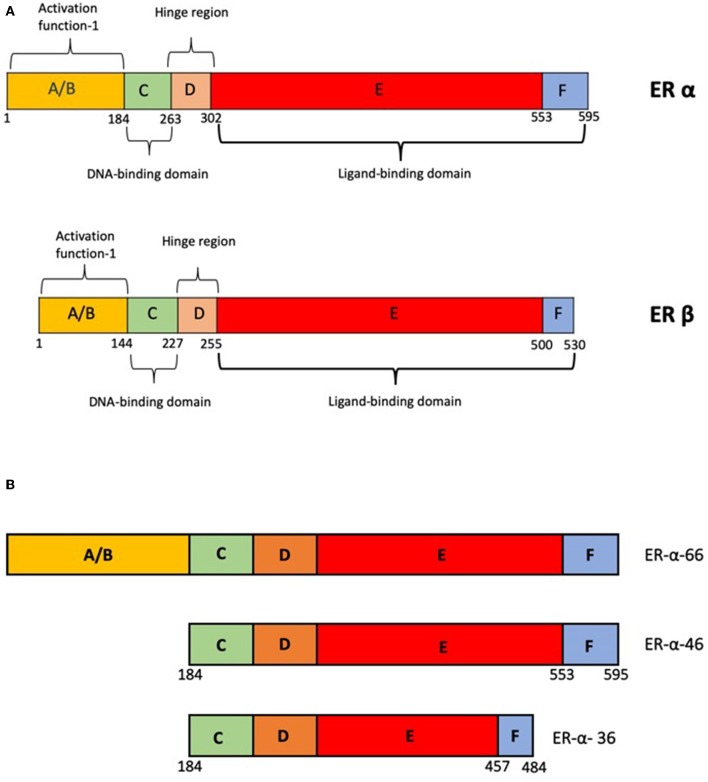
**(A)** Schematic representation of the domain structure of ER-α and ER-β. **(B)** Schematic representation of alternate variants of ER-α-products of alternate promoters.

Estrogen receptors, like other nuclear hormone receptors have a modular structure. The A and the B domains aid in binding to transcriptional regulators. The C domain aids in DNA binding and D forms the hinge region and also harbors the Nuclear Localization Signal, which helps in recognition and binding of specific DNA elements. E domain or the ligand binding domain confers ligand specificity (Figure 1A). In addition the E and F domains bind to additional co-regulators via the LXXLL motifs [reviewed in (2, 3)].

Classical estrogen signaling is mediated by two major receptors ER-α and ER-β. These two receptors are encoded by two distinct genes ESR-1 and ESR-2, respectively. The expression of these two genes vary in different tissues. ER-α has a dominant role in tissues such as uterus, mammary glands, pituitary, skeletal muscle, adipose, and bone; whereas, ER-β has a major role in ovary, prostate, lung, cardiovascular, and central nervous systems (4). Consistent with this, the knock-out mouse phenotypes of ER-α and ER-β are very different. While the ER-α KO are infertile with hypo-trophic uterus, the ER-β KO are sub-fertile and have reduced ovulation (5). There are still many aspects of ER-β actions and its role in pathophysiology of estrogen signaling including endocrine resistance that are largely unknown (3).

Estrogen receptors can bind to a variety of pharmacological agents which have either agonist-antagonist or only antagonist properties. These are referred to as SERMs and their effect is dependent on the target tissues. One example of this is tamoxifen which is used as an antagonist in breast cancer, but has agonistic effects on other estrogen target tissues. Raloxifene is anti-proliferative in breast but has a protective effect in the bone (6, 7). Fulvestrant, is a pure antagonist or Selective Estrogen Receptor Down-regulator (SERD). Both these groups of molecules bind to similar pockets in the ligand binding region of the ER. However, the conformational changes that are responsible for co-activator or co-repressor binding may be different. In the case of the SERMs, the conformations of the ER-α-LBD remain flexible and thereby responsible for the antagonist-agonist properties in a context dependent manner. However, in the case of SERD, the binding of ER-α to DNA is also affected (8–10) [reviewed in (3)].

## Alternative Forms of Estrogen Receptors

The most commonly found isoform of ER-α is a 66 kDa protein and is referred to as ER-α−66. Besides this, there are other forms of the receptor such as ER-α−36 and ER-α−46 (Figure 1B), which are located in the cytoplasm and plasma membrane in some tissues and breast cancer cell lines (11, 12). These proteins are products of alternative transcripts of the ESR-1 gene (13). There is no strong evidence to suggest the role of these truncated forms in any disease conditions. However, in the absence of full length receptor, these truncated receptors can mediate the rapid non-genomic actions by activating the EGFR/Src/ERK1/2 pathways (14).

Besides the alternative forms, the full length receptor also localizes to the plasma membrane and is responsible for the rapid actions of estrogen (15). The altered localization is due to differential post-translational modifications of ER-α and β (16). Mutation at Cys 447 disrupted the palmitoylation and also the ERK activation by estrogen further supporting the role of this modification in the non-genomic actions of estrogen (17). Palmitoylation on the ligand binding domains with the aid of HSP27, leads to interaction with caveolin 1 and transport to lipid rafts and cell membrane. The palmitoylated receptors dimerize within seconds of exposure to estrogen, and activate G-α and G βγ in a cell-type dependent manner (18). De-palmitoylation of the receptor leads to re-distribution of the protein and hence modulates the genomic and non-genomic actions also (19).

There are many mutations of the ESR1 gene reported in treatment refractory breast cancers. Amplification of ESR1 as well as gene fusions (ESR1/YAP, ESR1-CCDC170) are likely to be implicated in resistance, but the frequency of occurrence is fairly low (20, 21). On the other hand mutations clustered in the LBD have been reported in many endocrine resistant breast cancers (20–26). Advanced detection techniques such as droplet digital PCR and ctDNA analyses also have revealed that these mutations are seen mostly in advanced disease which have been exposed to endocrine therapies [reviewed in (27)]. Preclinical and clinical studies also suggest that these mutations probably exist in a small population of the tumor cells, which get selected under pressure of endocrine therapies and eventually the tumor emerges as endocrine resistant (28, 29).

The ESR1 mutations, which are mostly clustered in the LBD result in ER protein taking a conformation which is activated independent of ligand binding. These mutations were first described in the 1990s using structure-function studies in the absence of estrogen or the presence of antagonists. Y537 is a very commonly mutated site. This mutation makes the receptor constitutively active and results in reduced efficacy of anti-estrogens. L536, E380, and S463 are some of the other residues mutated in the ER. These mutations result the ER assuming an agonist conformation or reduce their affinities for co-repressors which result in constitutive activity. This subsequently results in insensitivity to endocrine therapies (30–32). K303 and S305 mutations have been found to affect sensitivity to endocrine therapies as well as cross-talk with other proliferative pathways such as IGF and Akt (33). A comprehensive review of these and other less frequent mutations and their structure-function implications is very thoroughly described in (34).

The mechanistic insight into the effects of these mutations on the function of the receptor and binding of ligands and antagonists has made it possible to strategize better drug designs and combinations. Increasing dosages, modifying side chains of the drugs for better affinity and potency are being tried. Besides, using ER degrading agents along with antagonists may also be a good strategy to combat endocrine resistance [reviewed in (34)].

Besides the variants of ER-α, there are other receptors such as the ERR gamma (estrogen-related receptor gamma), which play a role in resistance to tamoxifen. Studies on invasive lobular carcinomas (ILC) of the breast have shown that these resistant tumors have elevated levels of ERR gamma, and also increased AP-1 activation. This alternative signaling overcomes the growth inhibitory effects of tamoxifen rendering these tumors endocrine resistant (35, 36). Further, tamoxifen induces cell growth and this can be reverted by simultaneously inhibiting the FGF pathway. This study also suggests that ER drives a unique transcriptional program in these cells (37). This unique transcriptional response may be attributed to ERR gamma. ERR gamma was originally identified as an orphan receptor (38), which is constitutively active and can bind and regulate transcription from DNA elements such as steroidogenic factor 1 response element (SF-1RE) and estrogen response element (39). However, the affinity to these elements is variable. The transcriptional response of ERR gamma is quite different from the other members of this family such as ERR alpha and beta. While ERR alpha and beta constitutively activate transcription from ERE, TRE-Pal, and SF1-RE, it has been demonstrated that the activity of ERR gamma is dependent on the DNA element it binds to, which determines whether it associates with a co-activator such as PGC-1 (peroxisome proliferator-activated receptor gamma coactivator 1) or a co-repressor such as RIP140 (receptor interacting protein 140) (40, 41). These two regulators show a similar tissue distribution as ERR gamma (38, 42) and hence their relative levels may also affect ERR gamma activity. It is therefore suggested that these may be responsible for the unique gene expression pattern driven by ERR gamma (43).

## Estrogen Receptor β

The major actions of estrogen are mediated by two estrogen receptors ER-α and ER-β. The two proteins are coded for by separate genes located on chromosomes 6 and 14, respectively. Although ER-α and ER-β share a lot structural similarities (44) in terms of their domain organization (Figure 1A), there are differences in the ligand binding domain which makes the affinities for ligands different. Estrogen can activate both the receptors, but due to the spatio-temporal differences in the expression of the receptors, the biological effects may be different. There are sub-type selective agonists such as diarylpropionitrile which have been used in understanding these differences (45). The two receptors can form hetero-dimers which can bind DNA. The functionality of heterodimers in the presence of homodimers is not clear (46, 47). In many cases, ER-β antagonizes the effects of ER-α signaling. The knock-out phenotypes of the two receptors also suggest that the role of these two proteins may overlap to some extent but are more unique than similar (47). However, the exact mode of action of ER-β and its relevance in disease is less well-understood.

In the breast tissue, the number of cell-types expressing ER-β is more than ER-α (48). While ER-α is mostly localized to the epithelial cells, ER-β is also seen in the stroma (49). Studies on mouse models have revealed that while ER-α is responsible for proliferative effect of estrogen, ER-β is responsible for repressing proliferation and inducing apoptosis [reviewed in (50)]. Supporting this, there are many studies which have shown a correlation between increased ER-β levels with better disease free survival (51). Interestingly, in TNBC, which lacks ER-α, there is a subset of tumors that express ER-β. In these tumors, activating this pathway either with estrogen or a selective agonist inhibits cell proliferation by affecting the expression of several cell cycle related proteins thereby conferring a tumor suppressive role to ER-β (52).

ER-β levels vary in IDC and ILC. In a clinical trial it was observed that ILC shows better response to aromatase inhibitor treatment as compared to tamoxifen. This could be because, in the presence of ER-β, tamoxifen activates AP-1 site, and stimulates proliferation (53). Although other studies have shown a tumor suppressor action of ER-β, this study seems to indicate that the action depends on the context and the presence of certain co-factors including the presence of truncated ER-α.

Besides the expression of ER-β in tumor cells, there is evidence that several cells of the tumor microenvironment express the ERs to different extents. Among these, the immune cells are known to be affected by estrogen effects significantly (54). Studies have shown that a large percentage of TILs (tumor infiltrating leukocytes) express ER-β but not ER-α. Tamoxifen can act as an agonist for ER-β by activating AP-1, Sp1, and NF-κB sites. Therefore, tamoxifen treatment could have a significant impact on the immune surveillance. However, the exact modes of action and also the role of these leukocytes in tumor progression are yet to be characterized.

Considering the tumor suppressive roles of ER-β, and also the context dependent effects, it appears that selectively activating ER-β may be a good treatment strategy in breast cancer including overcoming resistance to tamoxifen. Many selective ER-β agonists are being tried in clinical trials [reviewed in (44)]. Also there are many natural sources of these agonists such as phyto-estrogens, isoflavones etc., which may hold promise in cancer prevention and treatment.

## Non-Nuclear Estrogen Receptor GPER-1

GPER-1 or GPR30 was originally classified as an orphan receptor and later identified as a non-nuclear estrogen receptor (55). In the subsequent years, it was demonstrated that GPER-1 was indeed a receptor estrogen and its natural ligand was 17-β estradiol (56). In fact, some studies have shown the effect of estrogen on cells which lack ER-α, suggested that some of the effects of estrogen which were originally believed to be mediated by ER-α, may in fact be mediated by GPER-1 (57).

GPER-1 is a G protein coupled receptor. It has an N-terminal extracellular domain and a C-terminal intracellular domain. Upon binding to 17-β-estradiol, it elicits a rapid and transient activation of many signaling pathways. There is an increase in cAMP production, intracellular calcium, synthesis of phosphotidyl inositol 3,4,5-triphosphate, transactivation of EGFR followed by activation of pathways such as PI3K-Akt and MAPK [reviewed in (58)]. Numerous functions have been attributed to GPR30 in a variety of cell types, which is supported by the knock out mouse phenotypes [reviewed in (59)].

The role GPER-1 in cancer is still unclear. In ER negative breast cancer cells, activating GPER-1 by an agonist such as G1, led to cell cycle arrest and apoptosis suggesting GPER-1 as a promising target for breast cancer therapy (59, 60). G-1 has also shown inhibition of proliferation in ER-α positive cell-lines (61). On the contrary, some studies show that in TNBC, GPER-1 is frequently over expressed and the growth of these tumors is driven by estrogen. Therefore, selectively blocking GPER-1 may be a good therapeutic approach (62). Mouse models show that mice lacking GPER-1 form smaller and histologically lower grade tumors compared to wild type mice (63). GPER-1 expression has been shown to be an indicator of poor prognosis in ovarian and endometrial cancers (64). It is also demonstrated that 17-β-estradiol can bind and activate this receptor and the genes that are activated are those involved in cell proliferation, migration etc. Blocking the GPR30 action using inhibitors such as calicosyn can inhibit proliferation in breast cancer cells (65). This is seen not only in cancer cells but also in the associated stromal cells (66). Tamoxifen and 4-hydroxy tamoxifen, which are the commonly used agents in endocrine therapy, can activate GPER-1. Once activated, this receptor further signals by activation of the G proteins, resulting in increased levels of cAMP and a series of events which leads to trans-activation of EGFR and activation of the ERK and MAPK pathways (67). Other agents used in endocrine therapy such as fulvestrant and raloxifene, which antagonize ER-α actions also act as agonists of GPER-1 and can promote tumor progression (68).

In the tumor microenvironment, GPER-1 activation also activates HIF-1-α dependent pathways aiding in angiogenesis and progression of breast cancer (69).

There are many studies which demonstrate the pro-tumorigenic effects of GPER-1 activation by tamoxifen, in a ER-α independent manner, which supports a role for GPER-1 in the development of resistance to endocrine therapies, particularly with SERMS [reviewed in (59)]. A study by Yu et al., has demonstrated that activation of GPER-1 and cytoplasmic localization in the stromal fibroblasts, activates the GPER/cAMP/PKA/CREB axis, which in turn triggers a metabolic switch in the CAFs. This switch provides the extra pyruvate and lactate to the tumor cells. This reprogrammed metabolism confers the tumor cells resistance to multiple therapeutics including tamoxifen, herceptin and chemotherapy (70). Targeting the GPER-1 or preventing the cytoplasmic localization may be a useful approach to retain drug sensitivity. Studies using selective agonists and antagonists for estrogen receptors, has made it more evident that GPER-1 has a major role to play in resistance to chemotherapy in ER positive breast cancers and also other cancers such as ovary and endometrium. Considering that the effects of GPER-1 is seen both in the tumors and in the microenvironment, targeting GPER-1 seems to be an attractive therapeutic strategy.

## Androgen Receptor

The androgen receptor has been seen to be expressed in a large percentage of breast tumors (71–75) including hereditary forms of breast cancer (76). Studies have shown that ER negative cancers do respond to AR blockade therapy suggesting a role for AR in prognosis (77, 78). It has also been demonstrated that tamoxifen resistant breast cancers show elevated expression of AR and this resistance can be reversed by AR antagonists such as bicalutamide (79). AR has also been implicated in resistance to aromatase inhibitors (80). Several studies have confirmed the role of AR in endocrine resistant breast cancers [(81) and reviewed in (82, 83)] making AR an important consideration in treatment of breast cancer.

**Figure 2:**
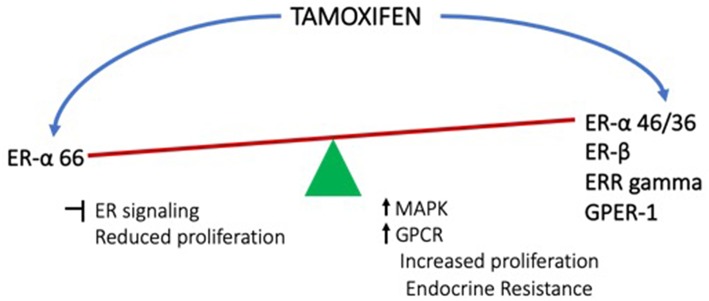
Effects of tamoxifen (endocrine therapy) on the conventional estrogen signaling from ER-α and the non-canonical signaling from other receptors.

Regardless of how it is initiated, the estrogen signaling from membrane can result in cell proliferation, altered metabolism, altered immune response as well as angiogenesis, all of which help in cancer cell survival, thereby promoting resistance. Considering the above discussed aspects of estrogen signaling and resistance to endocrine therapies, the following concerns emerge. Since estrogen is a major proliferative signaling pathway in cancers such as breast cancer, this has been the major target for therapy. Prior to discovery of other receptors, it was believed that the majority of actions of estrogen are mediated by ER-α and drugs targeting this receptor had been a choice strategy for therapy. Resistance to endocrine therapy was seen quite frequently, which was one of the major challenges. However, with the discovery of alternative forms of ER-α as well as other receptors for estrogen, non-canonical signaling has been gaining attention as one of the major causes of resistance. Since, the alternative receptors can be activated not only by estrogen, but also by some of the antagonists including tamoxifen, it becomes very important to understand the working of these receptors more comprehensively (Figure 2). This understanding may help in development of better therapeutic strategies. When and what determines the differential promotor usage for the variant isoforms of ER-α? How is the timing and extent of palmitoylation of the receptor controlled? How does the ER-α to ER-β ratio influence the treatment outcome and how can this be regulated? How does presence of GPER-1 influence the behavior of the tumor and its microenvironment in response to therapy? And what is the status of AR in the tumors? Combinatorial therapy definitely seems to hold better promise, but developing the right combinations is the herculean task. Future research needs to be focused on understanding the balance between the various modes of estrogen signaling and how this could be utilized optimally for better therapeutic outcome.

## Author Contributions

PR collected relevant literature and wrote the manuscript. NN and SN assisted in illustrations, formatting, and collection of literature.

### Conflict of Interest

The authors declare that the research was conducted in the absence of any commercial or financial relationships that could be construed as a potential conflict of interest.
